# MXSGD alleviates CsA-induced hypoimmunity lung injury by regulating microflora metabolism

**DOI:** 10.3389/fimmu.2023.1298416

**Published:** 2024-01-08

**Authors:** Chun Ye, Zi han Gao, Zi-yi Bie, Kai-qin Chen, Fang guo Lu, Ke Wei

**Affiliations:** Hunan University of Chinese Medicine, Hunan, China

**Keywords:** MXSGD, hypoimmunity, lung injury, CSA, flora metabolism

## Abstract

**Context:**

Ma Xing Shi Gan Decoction (MXSGD) is a traditional remedy for treating lung injuries that was developed by the Typhoid and Fever School of Pharmaceutical Biology. It has antitussive and expectorant effects, anti-inflammatory, antiviral, regulates the body’s immunity, etc.

**Aim:**

The aim of this study is to investigate whether MXSGD can ameliorate cyclosporine A (CsA)-induced hypoimmunity lung injury by regulating microflora metabolism. Methods: Establishment of a model for CsA-induced hypoimmunity lung injury. Using 16S rRNA high-throughput sequencing and LC-MS, the effects of MXSGD on gut flora and lung tissue microecology of mice with CsA-induced hypoimmunity were investigated.

**Results:**

MXSGD was able to preserve lung tissue morphology and structure, reduce serum inflammatory marker expression and protect against CsA-induced lung tissue damage. Compared to the model, MXSGD increased beneficial gut bacteria: *Eubacterium ventriosum group* and *Eubacterium nodatum group*; decreased intestinal pathogens: *Rikenellaceae RC9 intestinal group*; reduced the abundance of *Chryseobacterium* and *Acinetobacter*, promoted the production of *Lactobacillus* and *Streptococcus*, and then promoted the lung flora to produce short-chain fatty acids. MXSGD was able to enhance the expression of serum metabolites such as Americine, 2-hydroxyhexadecanoylcarnitine, Emetine, All-trans-decaprenyl diphosphate, Biliverdin-IX-alpha, Hordatin A and N-demethyl mifepristone in the CsA-induced hypoimmunity lung injury model.

**Conclusion:**

MXSGD can restore gut and lung microbiota diversity and serum metabolite changes to inhibit inflammation, ameliorate CsA-induced hypoimmunity lung injury.

## Graphical Abstract

Z: control; M: model; D: MXSGD; Red stands for an upward adjustment of the MXSGD after the intervention, and green for a downward adjustment.

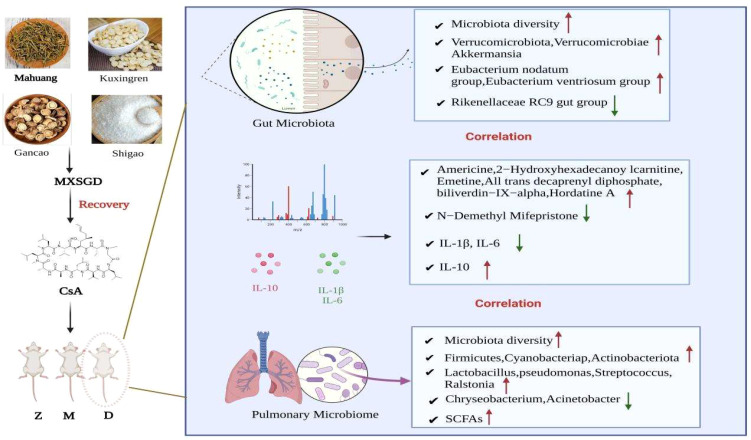

## 1 Introduction

Disruption of immune function due to viral infections, immunity inhibitors and other factors greatly increases the body’s susceptibility to pathogenic microorganisms and leads to infection of the lung tissue and other deeper tissues. Acute lung tissue injury caused by infection is the most important form of clinical pulmonary infectious disease (1, 2). Acute lung injury (ALI) is a common lung lesion and the synergistic action of various inflammatory mediators and immune cells can lead to a “storm of inflammatory factors” (3). Consequently, the body’s ability to maintain a stable state depends on the immune system’s ability to do its job.

Lung injuries have long been treated with traditional Chinese medicine, which has some distinct advantages. Ma Xing Shi Gan Decoction (MXSGD), which was derived from the ancient Chinese book Shang Han Lun, is a traditional prescription for the treatment of lung diseases, consisting of Gypsum Fibrosum (sulfates, Chinese name: Shigao), Glycyrrhiza uralensis (leguminosae, Chinese name: Gancao), Ephedra sinica (ehedraceae, Chinese name: Mahuang) and Semen armeniacae amarum (rosaceae, Chinese name: Kuxingren) (4). In China, these herbs have been used as traditional medicine for thousands of years. They have also been incorporated into other global systems of medicine that effectively relieve lung injuries (5). According to traditional Chinese medicine, Ma huang has anti-inflammatory properties, and Ku xing ren, Gan cao and Shi gao regulate the body’s immunity (6). In combination with these drugs, studies have shown that MXSGD is able to decrease the expression of inflammatory markers such as IL-6, IL-1β, TNF-α, etc., thus alleviating the severity of lung injury (7–10). MXGSD has been used for many centuries both for the prevention and treatment of respiratory diseases (11), However, the role of MXSGD in alleviating lung injury through microenvironmental flora and its metabolites, as well as its molecular mechanism, need to be further explored.

The metabolism of microflora has a significant impact on the body’s immune system and medication composition (12). Therefore, an essential technique to thoroughly investigate the curative effect and mechanism of traditional Chinese medicine is to systematically study the changes in metabolites and microecological flora. This is helpful in understanding the regulatory effect of drug molecules on the immunity of the organism. In this study, CsA established a model for lung tissue damage caused by hypoimmunity, and the mechanism of MXSGD to mitigate lung tissue damage was investigated in detail from the perspective of flora metabolism. These results served as an initial guide for further research on the value of traditional Chinese medicine in the prevention and treatment of lung tissue damage.

## 2 Experiment model and materials

### 2.1 Establishing the animal model and collecting samples

**Figure 1:**
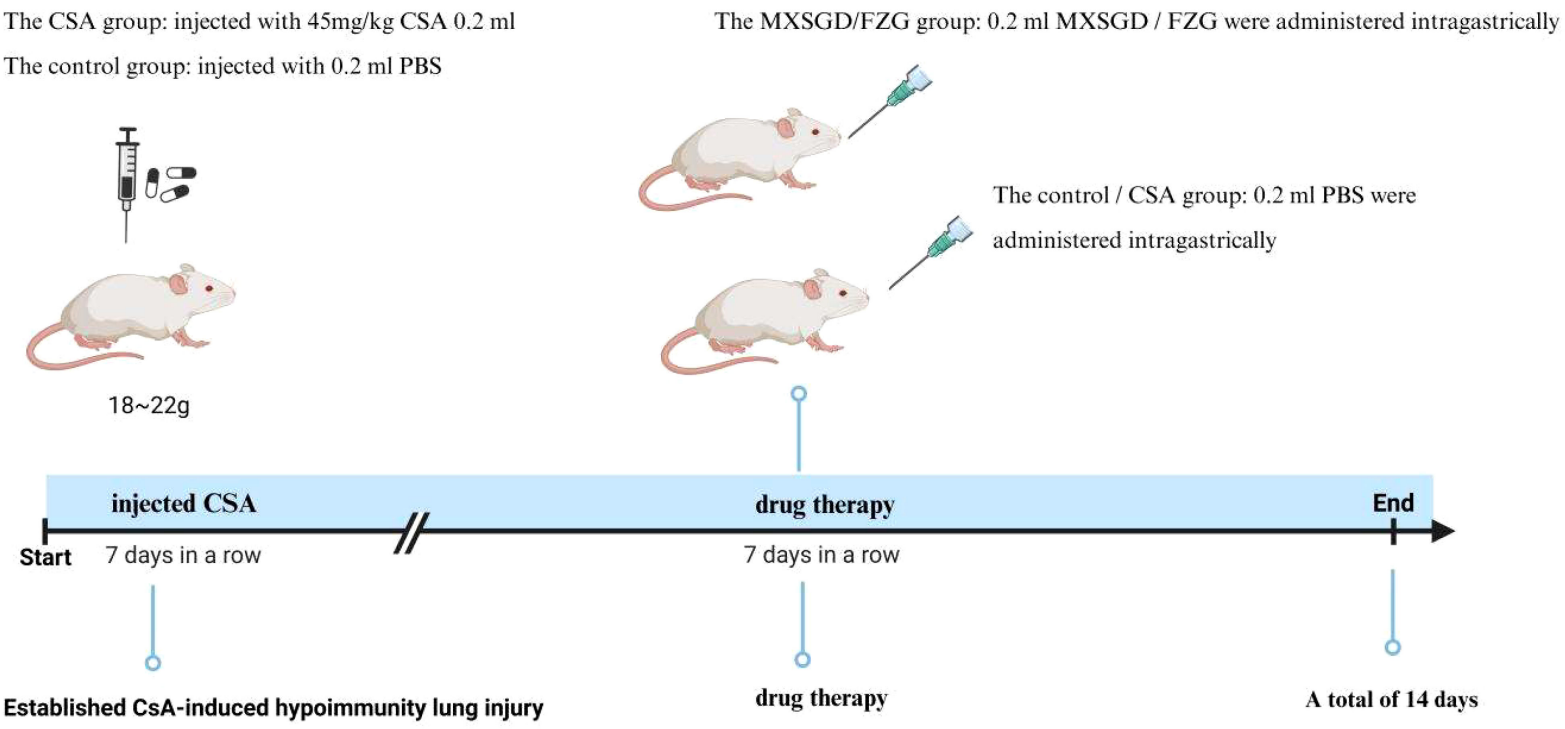
Establishing the animal model.

60 BLAB/c mice, 18~22g, half male and half female, SPF. Ethical number: LL20201202. After three days of adapted feeding, 60 mice were randomly divided into four groups: the control group (control), the model group (model), the Ma Xing Shigan decoction (MXSGD) and a fuzheng granule group (FZG), with 15 mice in each group. For 7 days, the hypoimmunity lung injury model was injected intraperitoneally with 45mg/kg CsA 0.2ml (13), while the control group was given the same dose of PBS. After 7 days, MXSGD and FZG were administered intragastrically for 7 days, while the other groups received the same amount of PBS (**Figure 1**). Mice were killed by cervical dislocation, peripheral blood was collected at 4°C and centrifuged at 3000rpm/15min, and the supernatant was frozen at -80°C for sample collection and follow-up experiments. Stool samples were collected from the cecum and kept in liquid nitrogen for 15 minutes and frozen at -80 degrees Celsius for later delivery. After 15 minutes in the liquid nitrogen solution, all lung tissue was removed and stored in the refrigerator at -80 degrees Celsius for later delivery and detection. All experimental designs used in the study were approved by the Hunan University of Chinese Medicine Ethics Committee. All procedures were conducted in accordance with the applicable rules and regulations.

### 2.2 Drugs

MXSGD consists of 9g of ephedra (batch number 2102260092), 9g of almond (batch number 2102260092), 18g of crude gypsum (batch number 211015) and 6g of grated licorice (batch number 211003) obtained from the outpatient pharmacy of the First Affiliated Hospital of Hunan University of Chinese Medicine. The amount of each dose of MXSGD was accurately weighed according to its composition. Ephedra 9g and 1L of high purity water were boiled for 25 minutes followed by 30min with gypsum, almonds and grilled liquorice. The second decoction residue is added to 500ml of water and boiled for 30min. After filtering, the drug solution is concentrated twice to 0.6g/ml. The positive control drug fuzheng granules were packed with composite film, 15g in each bag and 10 bags in each box (Chinese medicine Z20053398). It was diluted to 100mg/ml with PBS. CyclosporinA (CsA) 500mg (batch number HYM000082991) was diluted to 45mg/kg with PBS.

### 2.3 Organ index

The body weight and organ mass of the mice were measured, and the organ index was calculated. Organ index= organ mass (g)/body weight (g) ×100%.

### 2.4 Examination of lung hematoxylin-eosin staining

The collected lung tissues were used for hematoxylin-eosin (H&E) (Shanghai, China, Sinopharm Group Chemical Reagent Co., Ltd. Cat#10023418) staining analysis. The pathomorphological alterations in the lung tissues may be seen under a microscope through paraffin embedding, sectioning, and HE staining. In immunohistochemical staining, the experimental steps were performed strictly according to the instructions of the kit, DAB staining, hematoxylin post-staining, sealing tablets.

### 2.5 16S rRNA sequencing

After the mice were anaesthetised and executed, 2-3 pieces of faeces were quickly removed from the large intestine section with sterile forceps and placed in appropriately numbered sterile freezing tubes. Lung tissue samples were removed from the entire lungs of the mice with sterile forceps in sterile freezing tubes and immediately transferred to liquid nitrogen. Samples were collected in liquid nitrogen and after genomic DNA was extracted from the lung tissue/mouse faeces, the conserved region of rDNA was amplified using specific barcoded primers. The PCR amplification products were then cut, recovered and quantified using the QuantiFluorTM fluorometer. The purified amplification products were mixed in equal amounts, ligated with sequencing crosses to create sequencing libraries, and Illumina PE250 was used for online sequencing. (Guangzhou Genedenovo Biotechnology Co., Ltd for assistance with sequencing and/or bioinfomatic analysis).

### 2.6 LC-MS

After thawing the serum sample at room temperature and removing it from the refrigerator at -80°C, methanol-acetonitrile (V:V=2:1), a protein precipitating agent, was added in an amount of 300 ul and the mixture was swirled for one minute. Ultrasonication in an ice-water bath was adjusted to 40°C and centrifuged for 30 min; 4°C at 12000 rpm/10 min, and the supernatant of 200ul was placed in an LC-MS injection tube for evaporation. Throughout the experiment, a series of replicate data can be provided by injecting QC at regular intervals. Metabolites were analysed qualitatively using EMDB2.0, METLIN, Lipidmaps, Human Metabolome Database (HMDB) and other databases.

### 2.7 Measurement of Cytokine Concentrations

Appropriate amounts of lung tissue and serum were collected to analyse levels of interleukin-1(*IL-1β*), interleukin-6(*IL-6*) and interleukin-10(*IL-10*). Enzyme-linked immunosorbent assay (ELISA) was performed according to the instructions of the kit (Shanghai, China, enzyme-linked biology).

### 2.8 Analysis of mRNA Expression via Quantitative PCR (qPCR)

Lung tissue stored at -80°C was collected. Total RNA from each sample group was extracted according to the instructions of the total RNA extraction kit (lot number #218715, Beijing, China, Tiangen biochemical Technology Co., Ltd.) and cDNA (Jiangsu, China, lot number E096-01A Novoprotein Co., Ltd.) was synthesised by reverse transcription after removal of DNA. The cDNA was used as template and primers for *IL-6*, *IL-10* and *IL-1β* (**Table 1**) were added for amplification according to the NovoStart SYBR qPCR super mix. *β-Actin* was used as an internal reference and the relative expression of the mRNA was analysed by 2-_△△_Ct.

**Table 1:** Primer sequences for qRT-PCR.

Gene	Upstream primer	Downstream primer
*IL-1β*	5’-AAAGCTCTCCACCTCAATGG-3’	5’-CCCAAGGCCACAGGTATTT-3’
*IL-6*	5’-CTTCCATCCAGTTGCCTTCT-3’	5’-CTCCGACTTGTGAAGTGGTATAG-3’
*IL-10*	5’-TTGAATTCCCTGGGTGAGAAG-3’	5’-TCCACTGCCTTGCTCTTATTT-3’

### 2.9 Data processing

All data were analysed using the statistical programme SPSS 25. ANOVA was used to obtain a one-way approach for comparing the sample groups. Multiple comparisons were made with LSD when variances were aligned, while the Kruskal-Wallis rank sum test was used when variances were not aligned.

## 3 Results

### 3.1 Effect of MXSGD on mice infected with CsA

**Figure 2:**
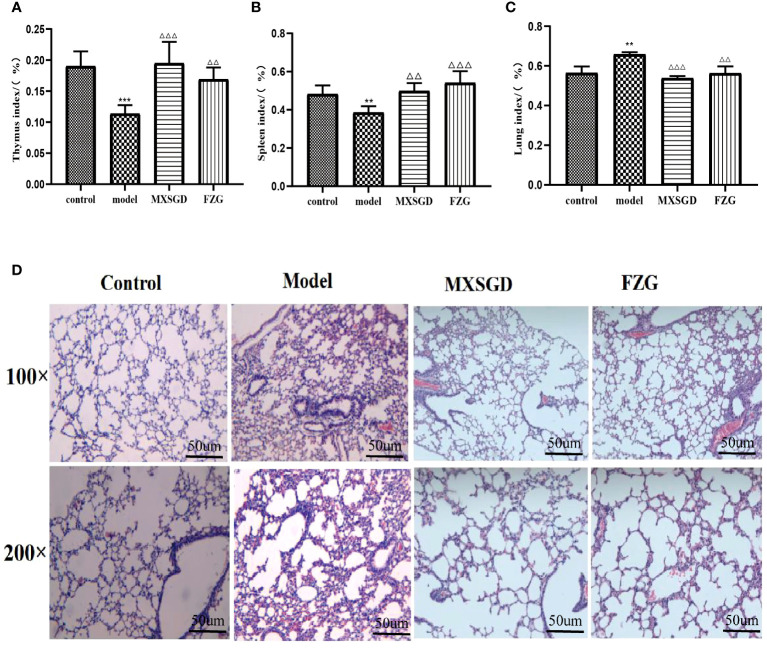
Effect of MXSGD on mice infected with CsA. **(A)** Thymus index(n>6); **(B)** Spleen index(n>6); **(C)** Lung index;(n>6); **(D)** Hematoxylin-eosin staining(HE)(n=3). Compared to the control, **p<0.01, and ***p<0.001; Compared to the model, △△p<0.01, and △△△p<0.001).

After 7 days of therapy with CsA alone, the thymus and spleen indices of the animals decreased compared to the control, showing that the hypoimmunity model induced by CsA was successful(**Figures 2A, B**). After 7 days, the thymus and spleen indices of MXSGD increased sharply compared to the model, and the index of FZG, a positive drug control group, increased significantly (*p*< 0.01). The result showed that the immunity of MXSGD was partially restored in mice. In addition, the lung index of the model increased dramatically compared to the control group, suggesting that CsA-induced hypoimmunity may influence lung tissue damage. After MXSGD therapy, the lung index improved (**Figure 2C**). To better understand the morphological changes in lung tissue, we investigated the inflammatory response in the lung induced by hypoimmunity after CsA infection and the protective effect of MXSGD on lung tissue damage. The lung tissue of the control mice was morphologically intact, with clear contours of the alveolar lumen and no obvious inflammatory cells in the lumen (**Figure 2D**), whereas in the model, alveolar collapse, hyperaemia and oedema, as well as infiltration of large numbers of lymphocytes and macrophages, indicated pathological injury to the lung tissue. The presence of inflammatory cells decreased, the alveolar wall thickened, the apoptotic consolidation space decreased and hyperaemia and oedema were reduced in MXSGD and FZG after treatment compared to control.

### 3.2 MXSGD improves the microecology of mouse lung tissue

**Figure 3:**
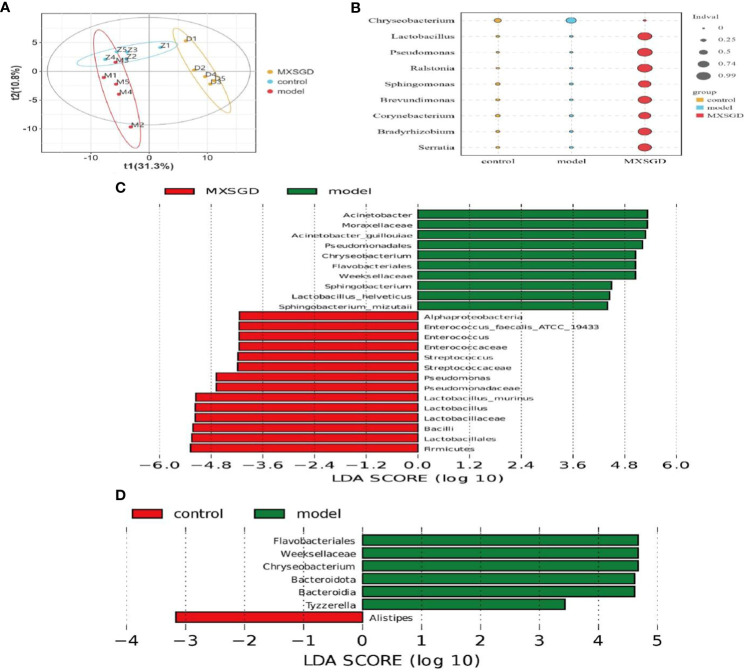
MXSGD improves the microecology of mouse lung tissue. Z: control; M: model; D: MXSGD; (Three groups, n=5) **(A)** Partial least squares-discriiminate analysis (PLS-DA). Abscissa: First principal component (t1); Ordinate: Second principal component (t2). Samples grouped differently are represented by different colours. The more similar the composition of the samples, the closer the samples are to each other graphically; **(B)** Indicator analysis (A biomarker is a species or community that indicates the environment in which it grows or certain environmental conditions in a particular area.) (*p*<0.01); **(C–D)** LEfSe analysis (LEFse first performs the Kruskal-Wallis rank sum test for all groups of samples and then compares the filtered differences between the two groups with the Wilcoxon rank sum test. The final filtered differences are ranked using the LDA (Linear Discriminant Analysis) results).

Alpha diversity (α-diversity) is mainly used to study the diversity of communities within a given biological environment (or in a sample) and can be assessed by calculating a range of α-diversity indices (e.g: Chao1, ACE, Shannon, Simpson, etc.) to assess the composition of the flora of a sample. Results of **Table 2** demonstrate that there the Shannon index, the Simpson index, the Chao index and the Ace index, were not statistically significant, indicating that the number of species in the lung flora of mice in each group and the number of individual species were not significantly different, i.e., the species richness and uniformity of the species of the lung flora in each group tended to be the same, so that the species structure and composition of the lung flora of each group and the comparison between the groups for β-diversity must be carried out. The *β*-diversity reflects the differences between different groups of samples. PLS-DA was used in the study to analyze the features of the lung flora in lung tissue samples taken from mice in each group, and Adonis analysis was pooled to detect significant differences. T1 and T2 were responsible for 31.3% and 10.8%, respectively, of the total abnormalities in the intestinal flora of the mice (**Figure 3A**). In addition, There is a significant change between the three groups of lung flora in mice, as demonstrated by indicator analysis (**Figure 3B**). With LEfSe Linear Discriminant Analysis (LDA > 4), we also examined the variations in flora between groups, which was used to evaluate the results of all categorisation levels (**Figures 3C, D**, **S1A, B**). Compared with the control, *Flavobacteriales*, *Weeksellaceae* and *Chryseobacterium* were mainly used as indicators in the model. After MXSGD intervention, indicator species such as *Firmicutes*, *Bacilli*, *Lactobacillus*, etc increased significantly, while the model mainly used *pseudomonadales*, *Moraxellaceae* and *Acinetobacter* as indicator species.

**Table 2:** α-diversity (x ± S, n=5).

Group	simpson	ace	shannon	chao
Control	0.62 ± 0.16	434.39 ± 61.49	2.40 ± 0.78	415.40 ± 52.30
Model	0.67 ± 0.08	414.80 ± 76.38	2.28 ± 0.40	395.63 ± 69.93
MXSGD	0.69 ± 0.22	387.33 ± 101.94	3.43 ± 1.63	375.61 ± 95.18

The correlation significance: p>0.05 between the three groups.

**Figure 4:**
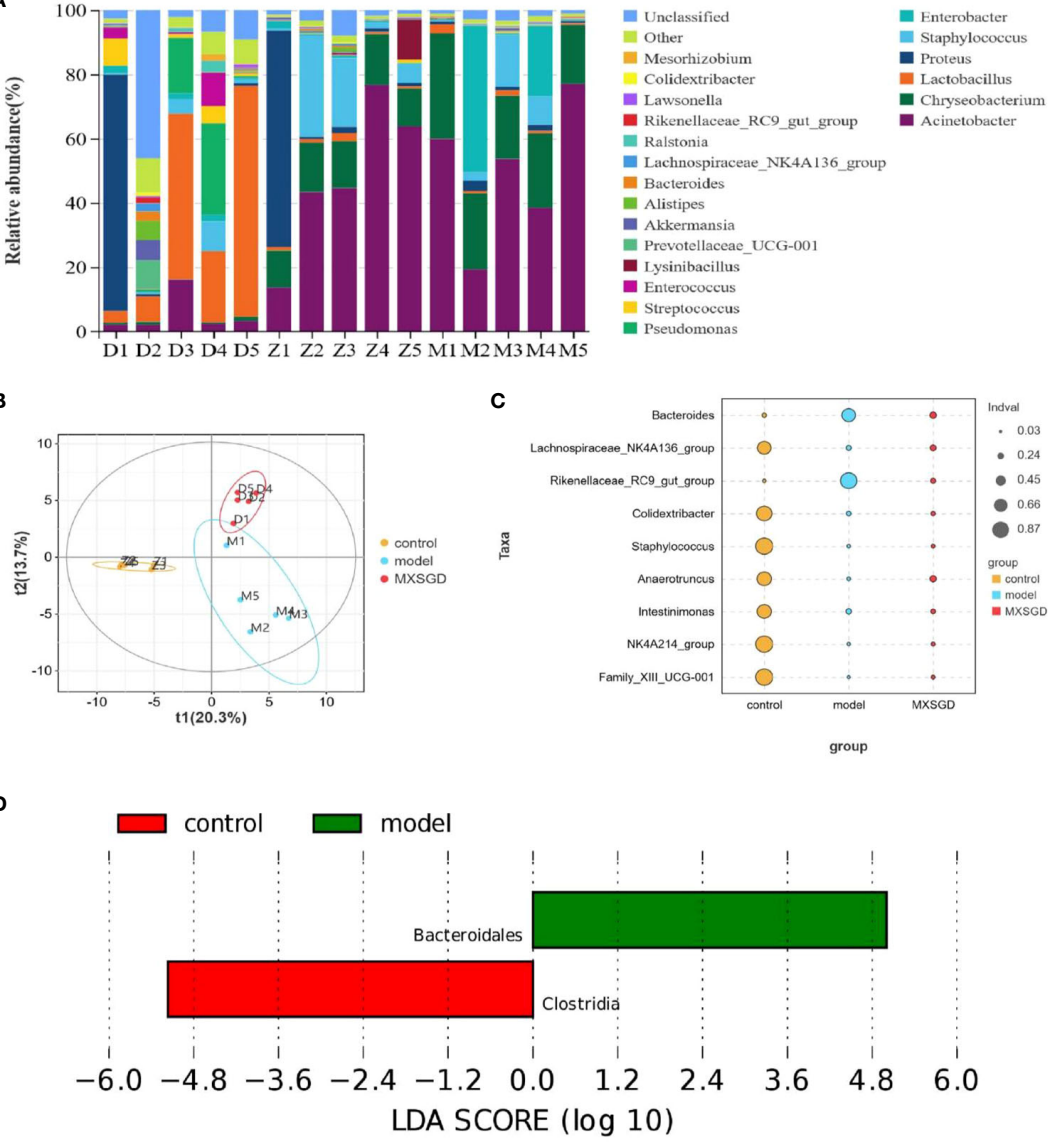
MXSGD improves the microecology of the Gut Microbiota. Z: control; M: model; D: MXSGD; (Three groups, n=5) **(A)** Top 20 differential metabolites at genus level; **(B)** PLS-DA; **(C)** Indicator analysis; **(D)** LEfSe analysis control VS model.

To further compare the differences in the species composition, we examined the 20 most abundant species at the phylum and genus levels, and we used heat maps to distinguish the microbial community at the genus (**Figure 4A**) and phylum (**Figure S2A**) levels. After MXSGD intervention, the *Firmicutes* (*p*=0.016), *Cyanobacteriap* (*p*=0.007), *Actinobacteriota* (*p*=0.007) and other species abundance was upregulated. At the genus level, The *Acinetobacter* (*p*=0.008) and *Chryseobacterium* (*p*=0.008) abundance both greatly increased in the model, whereas *Chryseobacterium* abundance (*p*=0.008) significantly declined after MXSGD intervention. The abundance of *Lactobacillus* (*p*=0.008), *pseudomonas* (*p*=0.03), *Streptococcus* (*p*=0.03) and *Ralstonia* (*p*=0.008) significantly increased after MXSGD intervention. The results show that MXSGD may improves the microecological of mouse lung tissue.

### 3.3 MXSGD improves the microecology of the gut microbiota

**Figure 5:**
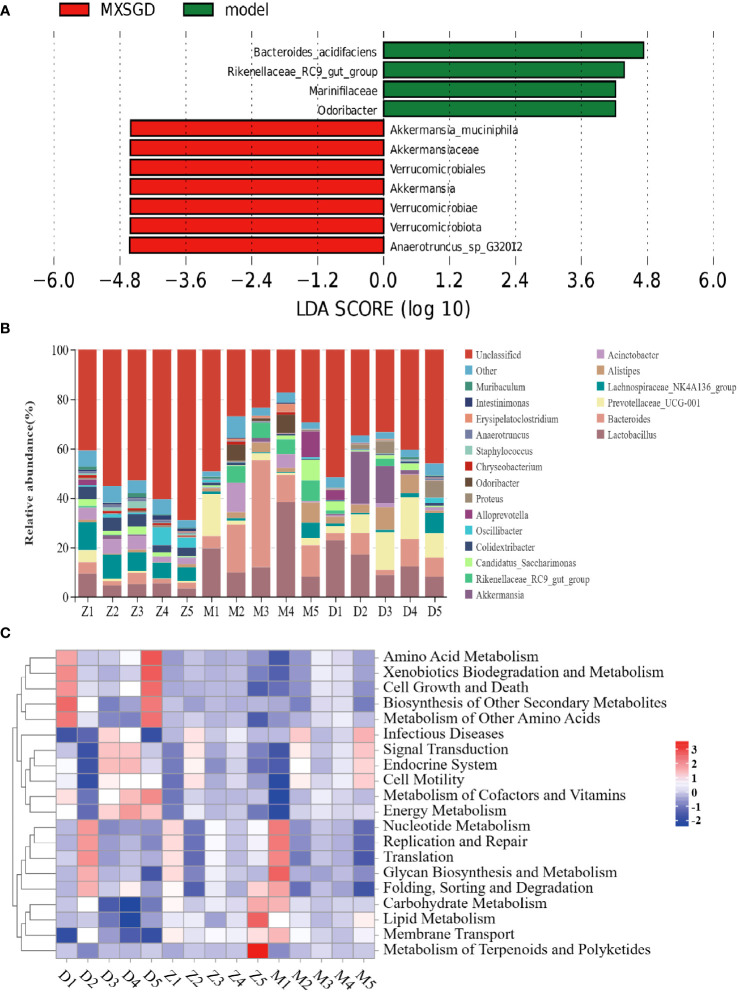
The composition and diversity of the gut microbiota are regulated by MXSGD. D: MXSGD; M: model; Z: control; (Three groups, n=5) **(A)** LEfSe analysis, Model VS MXSGD; **(B)** Top 20 differential metabolites at genus level in Gut Microbiota; **(C)** Heat map of Level 2 functional pathway of intestinal flora at genus level.

The dilution curve tends to be smooth, which suggests that the amount of sample sequencing data is reasonable based on the results of gut microbiota. Each sample of intestinal flora from them has an effective sequence of more than 80000. As can be seen in **Table 3**, the richness of the lung flora of the mice in all groups increased significantly (*P*< 0.05) compared to the control group after injection of different concentrations of CsA; there was no significant difference in diversity. The richness of the lung flora was significantly higher in each group compared to the control group (*P*<0.05); there was no significant difference in diversity. Then, the *β*-diversity PLS-DA analysis and Adonis test revealed significant changes in gut flora between the three groups, The structure of the intestinal flora of the model has changed compared to the control group. (*p*<0.003), which improved by the MXSGD intervention (Adonis, Bray–Curtismetric, *p*=0.001, R^2 ^= 0.2131), suggesting that MXSGD can regulate the structure of gut flora (**Figures 4B**, **S2B**). The results of the indicator analysis revealed that the gut flora of the control, model and MXSGD groups differed drastically (**Figure 4C**). To examine the results of all categorisation levels, we used LEfSe (linear discriminant analysis (LDA) > 5) to examine the differences in microflora between the groups (**Figures 4D**, **5A**, **S3A, B**). Compared with the control group, *Bacteroidales* is the most important indicators in the model. The primary indicators following MXSGD intervention were *Akkermansia, Verrucomicrobiae* and *Verrucomicrobiota* et al.

**Table 3:** α-diversity indexes (x ± S, n=5).

Group	simpson	chao	shannon	ace
Control	0.96 ± 0.01	872.07 ± 60.64	6.07 ± 0.23	902.21 ± 69.36
Model	0.91 ± 0.05	705.79 ± 75.00*	5.02 ± 0.47	726.96 ± 76.27*
MXSGD	0.94 ± 0.02	770.22 ± 99.06	5.31 ± 0.50	796.93 ± 94.65

Compared with the control, *P<0.05.

To further compare the differences in the species composition, we examined the 20 most abundant species at the phylum and genus levels, and we used heat maps to distinguish the microbial community at the genus (**Figure 5B**) and phylum (**Figure S2C**) levels. The abundance of *Bacteroidota* (*p*=0.01) at the level of phylum dramatically increased in the model as compared to the control, While *Firmicutes*, *proteobacteria*, *Verrucomicrobiota*, *patescibacteria*, *Actinobacteriota*, and *Campilobacterota*, saw a significant decline, but there was no statistical difference. The model’s abundance in *Rikenellaceae RC9 get group* (*p*=0.008), *Bacteroides* (*p*=0.017) and *Lactobacillus* (*p*=0.008) the increased dramatically at the genus level. Compared to the control, but significantly decreased in *Colidextribacter* (*p*=0.004). After the intervention of MXSGD, the abundance of *Rikenellaceae RC9 get group* was significantly downregulated, and the expression of *Eubacterium nodatum group* (*p*=0.03) and *Eubacterium ventriosum group* (*p*=0.01) was restored. These results thus suggest that MXSGD can improve the microecology of the gut microbiota.

### 3.4 Analysis to predict the metabolic function of the gut flora and the association of cytokines

**Figure 6:**
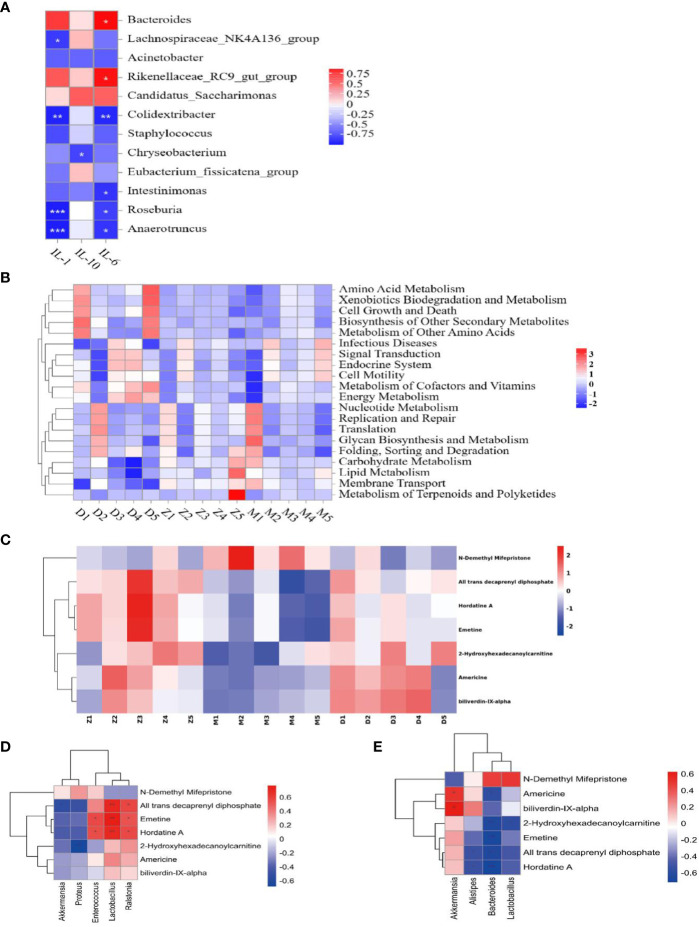
Gene function analysis is associated with inflammatory factors. Z: control; M: model; D: MXSGD; (Three groups, n=5) **(A)** Heat map of species correlation between intestinal flora and inflammatory factors; *IL-1: IL-1β*; The boxes show the statistical significance (^*^P<0.05,^**^P<0.01,^***^P<0.001); **(B)** Thermography of Level 2 functional pathway in lung tissue microecology at genus level; **(C)** Seven differential metabolites screened for LC-MS; **(D)** Heatmap of Spearman rank correlations for significantly different lung flora and metabolites. The boxes show the statistical significance (^*^P<0.05, ^**^P<0.01); **(E)** Heatmap of Spearman rank correlations for significantly different intestinal flora and metabolites. The boxes show the statistical significance (^*^P<0.05, ^**^P<0.01).

Based on the preliminary results, we performed Tax4 Fun analyses on the functional prediction of metabolites of the intestinal flora, as shown in the **Figure 5C**, and There are five different types of biological metabolic pathways, including environmental information processing, metabolism, cellular process, human disease and biological system. Compared with the control, the predicted gene abundance of intestinal microbiota in the model was significantly upregulated in metabolic regulation (energy metabolism, carbohydrate metabolism, metabolism of cofactors and vitamins, lipid metabolism, other amino acid metabolism, etc.), human diseases (immune diseasesn and eurodegenerative diseases), cell processing (cell communication), and other functional layer metabolic pathways (*p* < 0.05). To investigate the relationship between gut microflora and inflammatory factors, we analyzed the correlation “samplegut microflora-cytokines” with the Spearman correlation coefficient. At the genus level (**Figure 6A**), the *IL-1β* were negatively correlated with *Anaerotruncus* (*p*< 0.001), *Roseburia* (*p* < 0.001), *Colidextribacter* (*p*<0.01), *Lachnospiraceae_NK4A136_group* (*p*<0.05); the *IL-6* were negatively correlated with *Anaerotruncus* (*p*<0.05), *Roseburia* (*p*<0.05), *Intestinimonas* (*p*<0.05), *Colidextribacter* (*p*<0.01); but positively correlated with *Bacteroides* (*p*<0.05), *Rikenellaceae_RC9_gut_group* (*p*<0.05). The *IL-10* were negatively correlated with *Chryseobacterium* (*p*<0.05). It is clear that this intestinal flora may influences the expression and secretion of cytokines involved in immunity.

### 3.5 Analysis and prediction of the metabolic function of the pulmonary microflora

Based on the outcomes of the metabolite analyses of the lung flora, we made functional predictions with Tax4 Fun, as depicted in the **Figure 6B**, which includes six categories of related metabolic pathways: Metabolism, Genetic Information processing, Environmental Information processing, Cellular processes, Organismal Systems, and Human Disease, but the differences between groups were not significant.

### 3.6 Distribution of serum metabolites subjected to multivariate statistical analysis

**Figure 7:**
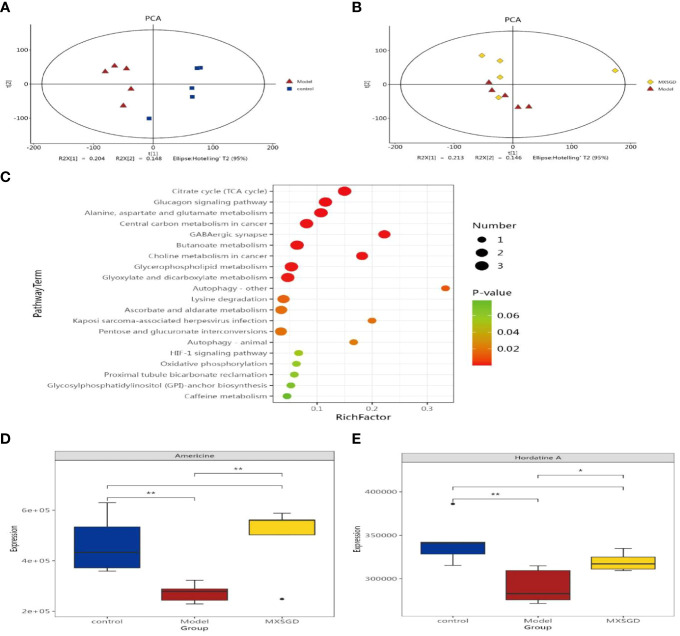
Detection of serum metabolites by LC-MS. **(A)** Principal Component Analysis (PCA) diagram between model and control; **(B)** OPLS-DA diagram between MXSGD and model; **(C)** Metabolic pathway enrichment bubble diagram(Different metabolic pathways were mapped using the KEGG pathway mapper function and the different metabolites were coloured according to the up- and down-regulation information. The small circles in the metabolic pathway map represent metabolites. The metabolites marked in red in the metabolic pathway map are experimentally detected upregulated metabolites, and the downregulated metabolites are blue); **(D)** Americine; **(E)** Hordatine A. The correlation significance: *p <0.05, **p <0.01.

To evaluate the serum metabolite changes in CsA-induced hypoimmunity lung tissue injury, serum samples were analyzed via LC-MS. The identification and selection of 2677 metabolites in the negative mode and 2235 metabolites in the positive mode for additional analysis. The fact that the metabolites of the model and control, MXSGD and model were essentially distinct from one another showed that the three groups’ metabolisms varied significantly (**Figures 7A, B**). By using LC-MS to identify 4912 metabolites in serum, the various metabolites were examined using the p-value<0.05, The Variable Importance in Projection (VIP) >1 standards. 197 differently expressing metabolites (DEM) were found in the model when compared to the control, and 136 DEM were shown to have significantly changed by MXSGD when compared to the model (**Figures S4A**). These distinct metabolites were mostly made up of organic acids and their derivatives, lipids and lipid-like molecules, benzenoids, organoheterocyclic compounds etc. Among these peaks, 114 metabolites were down-regulated in the model compared to the control, while 83 metabolites were up-regulated, 53 metabolites were up-regulated and 83 were down-regulated in the MXSGD compared with the model. KEGG pathway analysis indicated that most of the KEGG metabolic pathways, including the TCA cycle, glucagon signaling pathway, alanine, aspartate and glutamate metabolism, GABA-ergic synapse, glyoxylate and dicarboxylate metabolism, central carbon metabolism in cancer, choline metabolism in cancer, et al, were related to lipids and lipid-like molecular metabolites.

We discovered 136 metabolites had been significantly affected when compared the differences between the serum metabolites of MXSGD and the model. We categorized these metabolites owing to their relative abundance to gauge the close association between key DEM in light of the variety of their serum levels. The 20 types of DEM with the strongest correlation from LC-MS were selected as the subject of further research, provided that the correlation between the three group is significant. Compared with the control (**Figure 7C**), the model mainly up regulated 14 kinds of DEM, such as, phosphatidylserin(pS), lysophosphatidylcholine(Lyso), phosphatidylcholine(pC), bacterialureadiglucoside, phosphatidyl ethanolamine (pE) et al, as pE(19:0/20:2(11Z,14Z)), pS(18:1(9Z)/21:0), bacterio ruberidiglucoside, LysopC(20:4(8Z,11Z,14Z,17Z)/0:0)etc. However, the metabolites of LysopC(20:0/0:0), L−acetylcarnitineL, 14−methylpentadecanoylcarnitine14, 2−Hydroxyhexadecanoylcarnitine, Americine, Oxoglutaric acid showed a decreasing trend. Interestingly, among the 136 metabolites not screened, MXSGD upregulated the six metabolites (**Figure 6C**) Americine (**Figure 7D**), 2-Hydroxyhexadecanoy lcarnitine (**Figure S5A**), Emetine (**Figure S5B**), All trans decaprenyl diphosphate (**Figure S5C**), Biliverdin-IX-alpha (**Figure S5D**) and Hordatine A (**Figure 7E**) and downregulated the N-demethyl Mifepristone metabolite (**Figure S5E**). After screening the major DEM, we found that 10 metabolites showed an up-regulation. Some metabolites, including PC and Lyso, displayed a decreasing trend when MXSGD was present compared to the model group. For example: pC (17:0/15:1(9Z)), Clausarinol, N−demethylMifepristone, LysopC(0:0/18:2(9Z,12Z), PC(17:2(9Z,12Z)/0:0), LysopE (20:5(5Z,8Z,11Z,14Z,17Z)/0:0), [(2S,4R,5R,6R,14S,16R)−14−Hydroxy7,11dimethyl-6-(2-oxopyran-4-yl)-3-oxapentacyclo[8.8.0.02,4.02,7.011,16]octadecan−5−yl] acetate and other metabolites (**Figures S6A, B**). These findings imply that MXSGD may regulate metabolite alterations to mitigate the hypoimmunity damage to lung tissue caused by CsA.

### 3.7 Correlation analysis between the microflora and the various metabolites in serum

To further explore the correlation between differential metabolites in serum and flora, we correlated seven differential metabolites with flora at the level of the 20 most important genera by means of Spearman. Flora in the lungs (**Figure 6D**), the metabolites Emetine, All trans decaprenyl diphosphate and Hordatine A were positively correlated with *Lactobacillus*; Emetine and Hordatine A were positively correlated with *Enterococcus* and *Ralstonia*; 2-Hydroxyhexadecanoy lcarnitine was negatively correlated with *proteus*, et al. Trans decaprenyl diphosphate was negatively correlated with *Akkermansia*. Intestinal flora (**Figure 6E**), Metabolites Emetine, 2-Hydroxyhexadecanoylcarnitine, Hordatine A, All trans decaprenyl diphosphate were negatively correlated with *Bacteroides*. 2-Hydroxyhexadecanoy lcarnitine is negatively correlated with *Lactobacillus*; There was a negative correlation between All trans decaprenyl diphosphate, Hordatine A and alistipes. There was negative correlation between Americine, biliverdin-IX-alpha and Akkermansia. Thus, the interaction between serum metabolites and bacteria influences the body’s immune system.

### 3.8 MXSGD can reduces lung tissue inflammation

**Figure 8:**
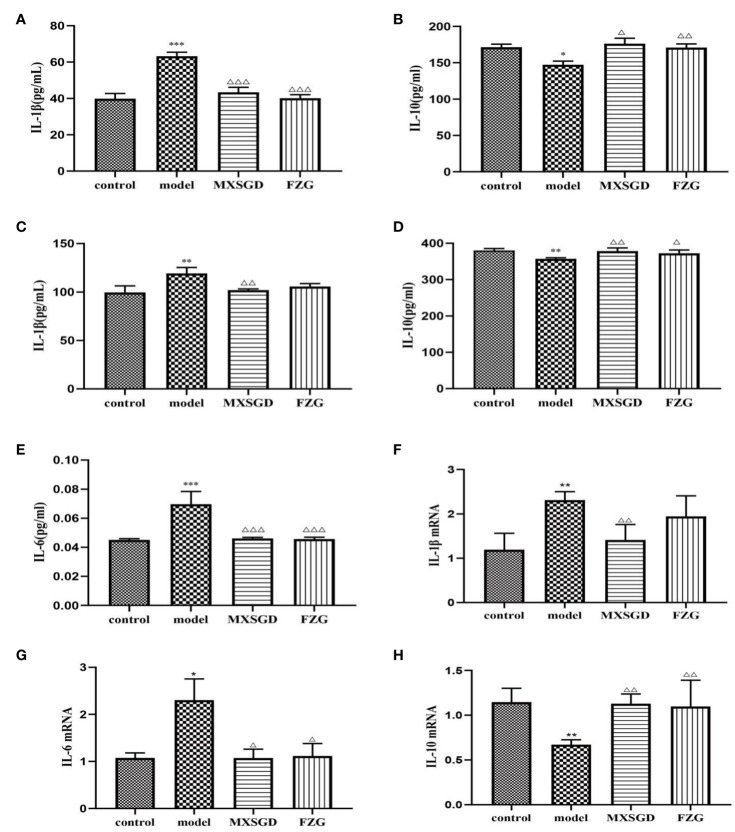
MXSGD can reduces lung tissue inflammation. **(A)** Mouse serum *IL-1β*; **(B)** Mouse serum *IL-10*; **(C)** *IL-1β* in lung tissue of mice; **(D)** *IL-10* in lung tissue of mice; **(E)** Mouse serum *IL-6*; **(F)** *IL-1β* mRNA gene expression; **(G)** *IL-6* mRNA gene expression; **(H)** *IL-10* mRNA gene expression; Compared to the control, ^*^p<0.05, ^**^p<0.01, and ^***^p<0.001; Compared to the model,^△^*p*<0.05, ^△△^*p*<0.01, and ^△△△^*p*<0.001).

To understand the affect of MXSGD on CsA-induced hypoimmunity in lung-injured mice, the expression levels of pro-inflammatory cytokine *IL-1β*, *IL-6* and anti-inflammatory cytokine *IL-10* were detected in serum and lung tissues. The results in serum showed that the level of pro-inflammatory cytokine *IL-1β* and *IL-6* dramatically increased and the level of anti-inflammatory cytokine *IL-10* considerably dropped after CsA infection (**Figures 8A, B, E**). After MXSGD intervention, The expression of *IL-1β* and *IL-6* in serum was markedly reduced while the expression of *IL-10* was increased. Consistent with the above results, MXSGD promoted the production of *IL-10* and decreased the expression of pro-inflammatory cytokine *IL-1β* in lung tissue (**Figures 8C, D**). Thus, it is possible that MXSGD protects ALI mice by reducing lung inflammation.

As can be shown, following CsA infection in mouse lung tissue, *IL-1β* and *IL-6* mRNA gene expression was dramatically increased (**Figures 8E–G**), while *IL-10* mRNA gene expression was markedly decreased (**Figure 8H**). *IL-1β* and *IL-6* mRNA gene expression were markedly decreased, while *IL-10* mRNA gene expression was increased by MXSGD therapy. Thus, our results imply that MXSGD protects against CsA-induced hypoimmunity lung injury.

## 4 Discussion

In this study, ELISA, RT-qPCR, HE, LC-MS and 16srRNA were used to investigate whether MXSGD ameliorates CsA-induced lung hypoimmunity by regulating microflora metabolism. The immune organ is the main site where the body performs immune functions. As the central immune organ, the spleen is an important peripheral immune organ of the body and a site where mature B and T cells settle, the thymus is the site where immune cells originate, differentiate, and mature (14). In this study, the spleen index and thymus index of the mice were calculated, and it was found that the indices decreased significantly after CsA modeling, indicating that the hypoimmunity mouse model was successfully established. Notably, the CsA-induced hypoimmunity lung injury was pronounced, It is characterised by a marked decrease in lung index, significant invasion of lung tissue by inflammatory cells, deterioration of alveolar structure, marked thickening of the alveolar wall, etc. The degree of inflammation and damage in the lungs of mice after MXSGD treatment was less pronounced, with clearer contours of the alveolar lumen and less infiltration of inflammatory cells in the lumen. Immune system function plays a critical role in homeostasis and is involved in the development of many diseases. Abnormalities in immune function have many unforeseen consequences, such as immunological dysfunction of the intestinal mucosa that can lead to diarrhoea in the host (15). Acute lung injury caused by lung infections due to immunodeficiency has become a major challenge in China’s (1). Pneumonia is aggravated by a dysregulation of the immune system caused by an alteration of the gut microbiota (16). Therefore, intestinal flora has recently emerged as a new target for the treatment of respiratory diseases. In this study, it was found that the intestinal flora of control mice at the phylum classification level is mainly composed of thick-walled bacteria, *Bacteroides*, *Verrucus*, *Microbacteria* and *Proteus*. Among them, the sum of the relative abundance of the first two doors is more than 90%, which occupies an absolutely dominant position. The abundance of *Firmicutes* (*p*=0.009) in the model decreased dramatically compared to the control. At the genus level, MXSGD increased the relative abundance of the *Eubacterium ventriosum* and *Eubacterium nodatum groups* and decreased the abundance of the *Rikenellaceae RC9 get group* compared to the model. Tax4 Fun function prediction results showed that the abundance of genes predicted by metabolic pathways related to lipid metabolism and immune diseases was reduced after MXSGD intervention, there was a correlation between gut flora and cytokines. These results suggest that MXSGD exerts its immunomodulatory effect by promoting the population of beneficial bacteria *Eubacterium ventriosum* and *Eubacterium nodatum*, while reducing the population of pathogenic *Rikenellaceae RC9 get group*.

The lung flora regulates the homeostasis of the lung immune system by maintaining the body’s immune tolerance to lung colonisation and dynamic balance during inflammatory infections (17). In the study, *Firmicutes* and *Proteobacteria* made up the majority of the microecology of normal lung tissue at 82%. The model is able to increase the abundance of *Chryseobactera* at the genus level. Research has shown that in people with a weakened immune system or infections of the lungs, the amount of *chrysobacteria* may be slightly increased (18). *Chryseobacterium* and *Acinetobacter* were drastically reduced after MXSGD intervention. MXSGD can promote the production of *Lactobacillus* and *Streptococcus*. *Lactobacillus* and *Streptococcus* are also part of the production of SCFAs (19), and *Lactobacillus* can inhibit the expression of *TNF-α* and prevent the occurrence of inflammation. Thus, by controlling lung microecology and SCFA production, MXSGD can prevent CsA-induced lung hypoimmunity. The amount and diversity of the lung microflora is less than that of the gut microbiota. There is no detectable change in the metabolism of the lung microflora because the change in the lung microflora is not as visible as the change in the gut microflora, but MXSGD also slightly mitigates the lung damage caused by CsA.

According to studies, organic acids can control the expression of inflammatory factors such as *IL-1β, IL-6* and *IL-10* in the host and enhance the immune response (20). The LC-MS results showed that Americine as an organic acid and its derivative showed a decreasing tendency under the conditions of hypoimmunity and that MXSGD therapy could promote the formation of this metabolite and improve the immune system response. Inflammation-related damage in the lung is mainly caused by the metabolism of glycerol, a phospholipid that is a component of the cell membrane (21, 22). Studies have shown that the model of lung injury in mice with hypoimmunity induced by CsA had the largest and most significant fraction of glycerol phospholipid metabolites. LysopC (20:5(5Z,8Z,11Z,14Z,17Z)/0:0), LysopC (20:4 (8Z,11Z,14Z,17Z)/0:0), and LysopC (22:6(4Z,7Z,10Z,13Z,16Z,19Z)/0:0) were significantly increased in the model. The hypothesis is that glycerol-phospholipid metabolites might increase inflammation in ALI by altering lipid metabolism. Lyso could play a role in the inflammatory process (23). MXSGD intervention was able to regulate part of LysopE (20:5(5Z,8Z,11Z,14Z,17Z)/0:0) and LysopC(0:0/18:2(9Z,12Z)) and then alleviate the disruption of glycerol phospholipids. In our study, 75 different lipids were detected to have changed, representing 55% of all different metabolites. Therefore, we speculate that MXSGD may prevent CsA-induced lung hypoimmunity by controlling the formation of lipids and lipid-like molecular metabolites such as glycerol phosphate metabolites. Of these differential metabolites, 2-Hydroxyhexadecanoylcarnitine, Emetine, All-trans-decaprenyl Diphosphate, Americine, Biliverdin−IX−alpha, Hordatin A and N-demethyl-mifepristone metabolites were significantly altered after MXSGD intervention. However, as complete KEGG pathway information is not available for these metabolites, they do not appear in the database. Based on the interactions between seven classes of metabolites and changes in bacterial flora, we discovered that these metabolites were mainly associated with the bacteria *Bacteroides*, *Lactobacillus*, *Akkermansia*, *Alistipes*, *Enterococcus*, *Ralstonia* and *Akkermansia*. *Akkermansia* has been reported to improve the integrity of intestinal epithelial cells and the thickness of the mucus layer, and to promote the intestinal health of the host (24). *Bacteroides* activates PPAR-γ to regulate immunity by degrading polysaccharides and producing butyric acid (25). The dysbiotic microbiota promotes the proliferation of opportunistic pathogens, reduces the abundance of commensal bacteria and leads to damage to the microbiota that colonises the microbiota. In fact, the symbiotic microbiota plays an important role in maintaining the integrity of the barrier by producing beneficial metabolites such as SCFAs. There is a complex and close interaction between serum metabolites and microorganisms. Microorganisms can affect the body’s immune system and thereby indirectly influence the synthesis and degradation of serum metabolites. Some studies have shown that the microbial community in the human body is closely related to the development and function of the immune system. Changes in the microbiota can lead to disturbances in the immune system, which in turn affect the normal function of serum metabolites (26, 27). If we know the KEGG pathway in which these metabolites are found, we can examine more closely the mechanism of correlation between these metabolites and the microbial communities.

The inflammatory response is an significant mechanism of the immune defence that regulates ALI in the body(CHEN Kaiqin and al, 2022). After treatment with MXSGD, levels of *IL-6* and *IL-1β* decreased dramatically in serum of mice with CsA-induced lung injury, while levels of *IL-10* were significantly increased. MXSGD can also reduce CsA-induced lung injury by decreasing the expression of *IL-6* and *IL-1β* mRNA. MXSGD can therefore be considered a suitable drug for the treatment of lung damage caused by CsA-induced hypoimmunity.The results of the correlation analysis between intestinal flora and cytokines showed that the content of the Pro-inflammatory cytokine *IL-6* and *IL-1β* were favorably connected with *Roseburia, Intestinimonas* and *Lachnospiraceae NK4A136 group*, but negatively correlated with *Bacteroides and Rikenellaceae_RC9_gut_group*, *IL-10* were negatively correlated with *Chryseobacterium.* according to this study’s investigation of the relationship between gut flora and cytokines. The *Lachnospiraceae_NK4A136_group* is one of the most important butyrate-producing bacteria whose presence can improve intestinal barrier function in rats, and whose presence is significantly negatively correlated with the degree of inflammation (16, 28). Studies have shown that *Lachnospiraceae NK4A136 group* is a conditional pathogen highly related to intestinal flora imbalance (29, 30). *Roseburia* as an anti-inflammatory factor has a protective effect against systemic inflammation caused by dysbiosis of the intestinal microbiota (31). Thus, MXSGD may increase the diversity of the gut microbiota and alter the relative abundance of bacteria to attenuate gut dysbiosis in mice with CsA-induced hypoimmunity, attenuate the inflammatory response and promote immunomodulatory effects. We believe that reconfiguration of the gut microbiota as a result of MXSGD contributes to the healing and recovery of CsA-induced hypoimmunity lung injury.

In summary, MXSGD attenuates CsA-induced hypoimmunity lung tissue injury. Its mechanism may be to restore gut and lung microbiota diversity and serum metabolite changes to prevent inflammation. However, this study did not investigate how MXSDG regulates interactions between microenvironmental flora and metabolites in serum, which will be further explored by this group.

## Data availability statement

The original contributions presented in the study are publicly available. The LC-MS data presented in the study are deposited in the OMIX repository, accession number OMIX005419.The 16srRNA data presented in the study are deposited in the GSA repository, accession number CRA014039 and CRA014038.

## Ethics statement

The animal study was approved by Ethical review of animal experiments in Hunan University of Traditional Chinese Medicine. The study was conducted in accordance with the local legislation and institutional requirements.

## Author contributions

CY: Writing – original draft. ZG: Data curation, Writing – review & editing. K-QC: Software, Writing – review & editing. Z-YB: Formal Analysis, Writing – review & editing. FL: Conceptualization, Writing – review & editing. KW: Writing – review & editing.

## References

[1] Azoulay E Russell L Van de Louw A Metaxa V Bauer P Povoa P. Diagnosis of severe respiratory infections in immunocompromised patients. Intensive Care Med (2020) 46(2):298–314. doi: 10.1007/s00134-019-05906-5 32034433 PMC7080052

[2] Ye C Gao ZH Chen KQ Lu FG Wei K. Research on pachymaran to ameliorate csA-Induced immunosuppressive lung injury by regulating microflora metabolism. Microorganisms (2023) 11(9):2249. doi: 10.3390/microorganisms11092249 37764093 PMC10537689

[3] Ling LI. Effect of Maxing Shigan Decoction against type A influenza virus infection in mice induced by viral lung injury based on TLR4-MyD88-TRAF6 signal pathways. Chin Traditional Herbal Drugs (2017) 48(8):1596. doi: 10.7501/j.issn.0253-2670.2017.08.017

[4] Li M Fan X Zhou L Jiang M Shang E. The effect of Ma-Xin-Gan-Shi decoction on asthma exacerbated by respiratory syncytial virus through regulating TRPV1 channel. J Ethnopharmacol (2022) 291:115157. doi: 10.1016/j.jep.2022.115157 35247474

[5] Nose M Kobayashi R Tada M Hisaka S Masada S Homma M. Comparison of ephedrine and pseudoephedrine contents in 34 Kampo extracts containing Ephedrae Herba used clinically in Japan. J Nat Med (2023) 77(3):476–88. doi: 10.1007/s11418-023-01687-w 36854954

[6] Zhang Y Liu X Xue Z Feng W Zhang B Zhang Lei. Research progress in prescriptions of maxing shigan decoction. Modern Anim Husbandry Sci Technol (2023) (01) 5–8. doi: 10.19369/j.cnki.2095-9737.2023.01.002

[7] Zhang S-y He G-l Lu F-g Li L Zhang B Dai B. Mechanism research of anti influenza virus of ephedra decocted earlier Maxing Shigan Decoction from the expression level of IFN-α/β protein mediated by TLR7/8. Chin J Traditional Chin Med (2019) 34(03):1193.

[8] Xi L Le-Ping L Xin-Yi XU. A network pharmacology study on the effects of ma xing shi gan decoction on influenza. Digital Chin Med (2020) 13(3):1348–59. doi: 10.1016/j.dcmed.2020.09.003

[9] Guo T Guo Y Liu Q Xu Y Wei L Wang Z. The TCM prescription Ma-xing-shi-gan-tang inhibits Streptococcus pneumoniae pathogenesis by targeting pneumolysin. J Ethnopharmacol (2021) 275:114133. doi: 10.1016/j.jep.2021.114133 33892068

[10] Shi Y Luo J Narbad A Chen Q. Advances in lactobacillus restoration for β-Lactam antibiotic-Induced dysbiosis: A system review in intestinal microbiota and immune homeostasis. Microorganisms (2023) 11(1):179. doi: 10.3390/microorganisms11010179 36677471 PMC9861108

[11] Hsieh CF Lo CW Liu CH Lin S Yen HR Lin TY. Mechanism by which ma-xing-shi-gan-tang inhibits the entry of influenza virus. J Ethnopharmacol (2012) 143(1):57–67. doi: 10.1016/j.jep.2012.05.061 22710290

[12] Wang Z Xie J Yang Y Zhang F Wang S Wu T. Sulfated Cyclocarya paliurus polysaccharides markedly attenuates inflammation and oxidative damage in lipopolysaccharide-treated macrophage cells and mice. Sci Rep (2017) 7:40402. doi: 10.1038/srep40402 28094275 PMC5240341

[13] Kaiqin C Ke. W Chun YE Tianhao Z Bo Z Rong X. Immunomodulatory effect of pachymaran on cyclosporine A (CsA)-induced lung injury in mice. Digital Chin Med (2022) 5(02)(02):232. doi: 10.1016/j.dcmed.2022.06.011

[14] Parkin J Cohen B. An overview of the immune system. Lancet (2001) 357(9270):1777–89. doi: 10.1016/S0140-6736(00)04904-7 11403834

[15] Ding S Jiang H Fang J. Regulation of immune function by polyphenols. J Immunol Res (2018) 2018:1264074. doi: 10.1155/2018/1264074 29850614 PMC5925142

[16] Wang P Gao J Ke W Wang J Li D Liu R. Resveratrol reduces obesity in high-fat diet-fed mice via modulating the composition and metabolic function of the gut microbiota. Free Radic Biol Med (2020) 156:83–98. doi: 10.1016/j.freeradbiomed.2020.04.013 32305646

[17] Lloyd CM Marsland BJ. Lung homeostasis: influence of age, microbes, and the immune system. Immunity (2017) 46(4):549–61. doi: 10.1016/j.immuni.2017.04.005 28423336

[18] Starovoĭtova SO Tymoshok NO VIu H MIa S. [Immunomodulation characteristics of Lactobacillus genus bacteria]. Mikrobiolohichnyi zhurnal (Kiev Ukraine 1993) (2009) 71(3):41–7. 19938605

[19] Ebeid TA Al-Homidan IH. Organic acids and their potential role for modulating the gastrointestinal tract, antioxidative status, immune response, and performance in poultry. World's Poultry Sci J (2022) 78(1):83–101. doi: 10.1080/00439339.2022.1988803

[20] Fessler MB Summer RS. Surfactant lipids at the host-environment interface. Metabolic sensors, suppressors, and effectors of inflammatory lung disease. Am J Respir Cell Mol Biol (2016) 54(5):624–35. doi: 10.1165/rcmb.2016-0011PS PMC4942198 26859434

[21] Murakami Y Hoshi M Hara A Takemura M Arioka Y Yamamoto Y. Inhibition of increased indoleamine 2,3-dioxygenase activity attenuates Toxoplasma gondii replication in the lung during acute infection. Cytokine (2012) 59(2):245–51. doi: 10.1016/j.cyto.2012.04.022 22609210

[22] Li L Fu W Wu R Song Y Wu W Yin S. Protective effect of Ganoderma atrum polysaccharides in acute lung injury rats and its metabolomics. Int J Biol Macromol (2020) 142:693–704. (C). doi: 10.1016/j.ijbiomac.2019.10.010 31739063

[23] Zhang T Li Q Cheng L Buch H Zhang F. Akkermansia muciniphila is a promising probiotic. Microb Biotechnol (2019) 12(6):1109–25. doi: 10.1111/1751-7915.13410 PMC6801136 31006995

[24] Wexler AG Goodman AL. An insider's perspective: Bacteroides as a window into the microbiome. Nat Microbiol (2017) 2:17026. doi: 10.1038/nmicrobiol.2017.26 28440278 PMC5679392

[25] Du L Zhang J Zhang X Li C Wang Q Meng G. Oxypeucedanin relieves LPS-induced acute lung injury by inhibiting the inflammation and maintaining the integrity of the lung air-blood barrier. Aging (2022) 14(16):6626–41. doi: 10.18632/aging.204235 PMC9467393 35985771

[26] Zhang X Yang Y Su J Zheng X Wang C Chen S. Age-related compositional changes and correlations of gut microbiome, serum metabolome, and immune factor in rats. Geroscience (2021) 43(2):709–25. doi: 10.1007/s11357-020-00188-y PMC8110635 32418021

[27] Fan Y Gao Y Ma Q Yang Z Zhao B He X. Multi-omics analysis reveals aberrant gut-metabolome-immune network in schizophrenia. Front Immunol (2022) 13:812293. doi: 10.3389/fimmu.2022.812293 35309369 PMC8927969

[28] Sheng K Yang . J Xu Y Kong X Wang J Wang. Y. Alleviation efects of grape seed proanthocyanidin extract on infammation and oxidative stress in a D-galactose-induced aging mouse model by modulating the gut microbiota. Food Funct (2022) 13(3):1348–59. doi: 10.1039/D1FO03396D 35043135

[29] Tan JY Tang YC Huang J. Gut microbiota and lung injury. Adv Exp Med Biol (2020) 1238:55–72. doi: 10.1007/978-981-15-2385-4_5 32323180

[30] Swolana D Wojtyczka RD. Activity of Silver Nanoparticles against Staphylococcus spp. Int J Mol Sci (2022) 23(8):4298. doi: 10.3390/ijms23084298 35457115 PMC9028791

[31] Zhao C Bao L Qiu M Wu K Zhao Y Feng L. Commensal cow Roseburia reduces gut-dysbiosis-induced mastitis through inhibiting bacterial translocation by producing butyrate in mice. Cell Rep (2022) 41(8):111681. doi: 10.1016/j.celrep.2022.111681 36417859

