# Supplementary material for: MXSGD alleviates CsA-induced hypoimmunity lung injury by regulating microflora metabolism
Source: Front Immunol. 2024 Jan 8;14:1298416. doi: 10.3389/fimmu.2023.1298416 (PMC10801022; doi:10.3389/fimmu.2023.1298416)
Supplement: Supplementary file 1 [file DataSheet_1.pdf]

Supplementary

materials:

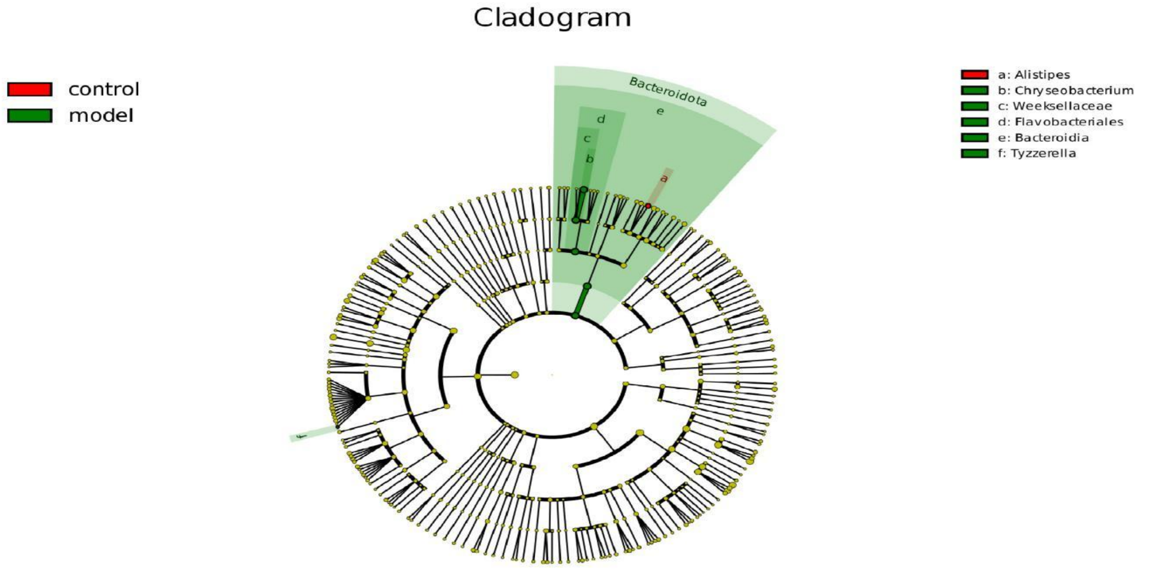

A

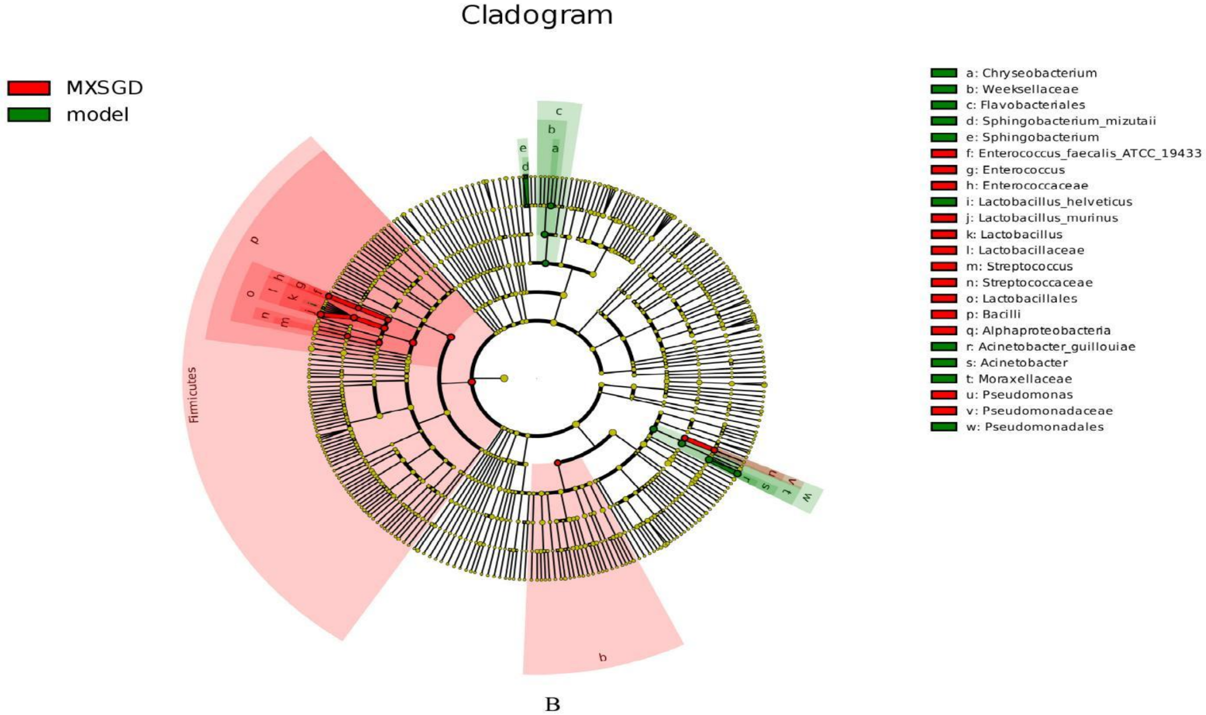

B

Fig. 1 MXSGD improves the Microecological of mouse lung tissue

(**A**): LEfSe analysis, control VS model ; (**B**): LEfSe analysis, MXSGD VS model

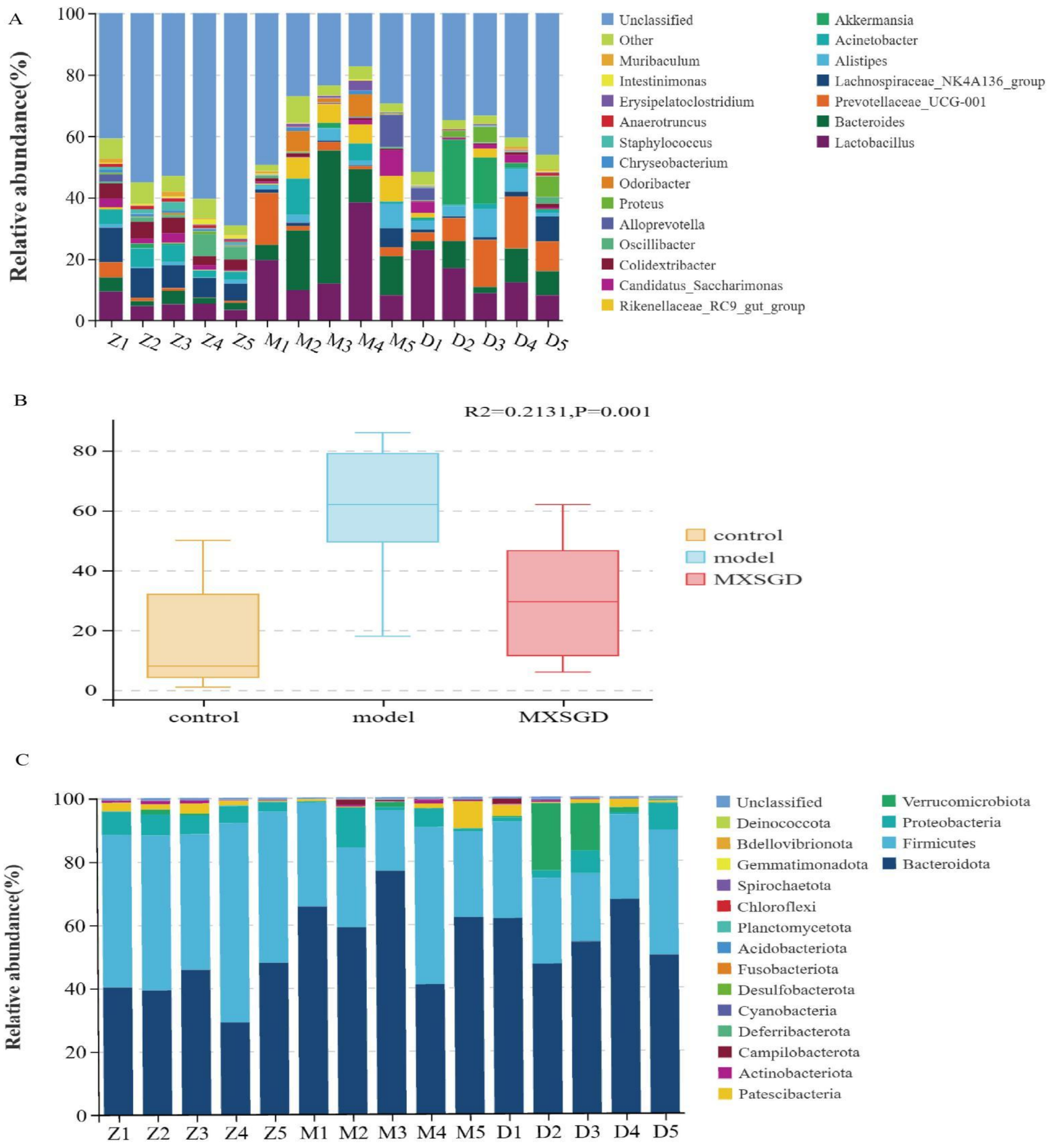

Fig.2 MXSGD Regulates the Diversity and Composition in mice

(**A**):Species distribution map of the microbial community at the phylum level in lung microecology; (**B**):The Adonis test, also called Permutation Multivariate Analysis of Variance

(Permanova) or Nonparametric Multivariate Analysis of Variance (Nonparametric MANOVA), is a commonly used unsupervised multivariate analysis of variance.(**C**):Species distribution map of the microbial community at the phylum level in Gut Microbiota

A

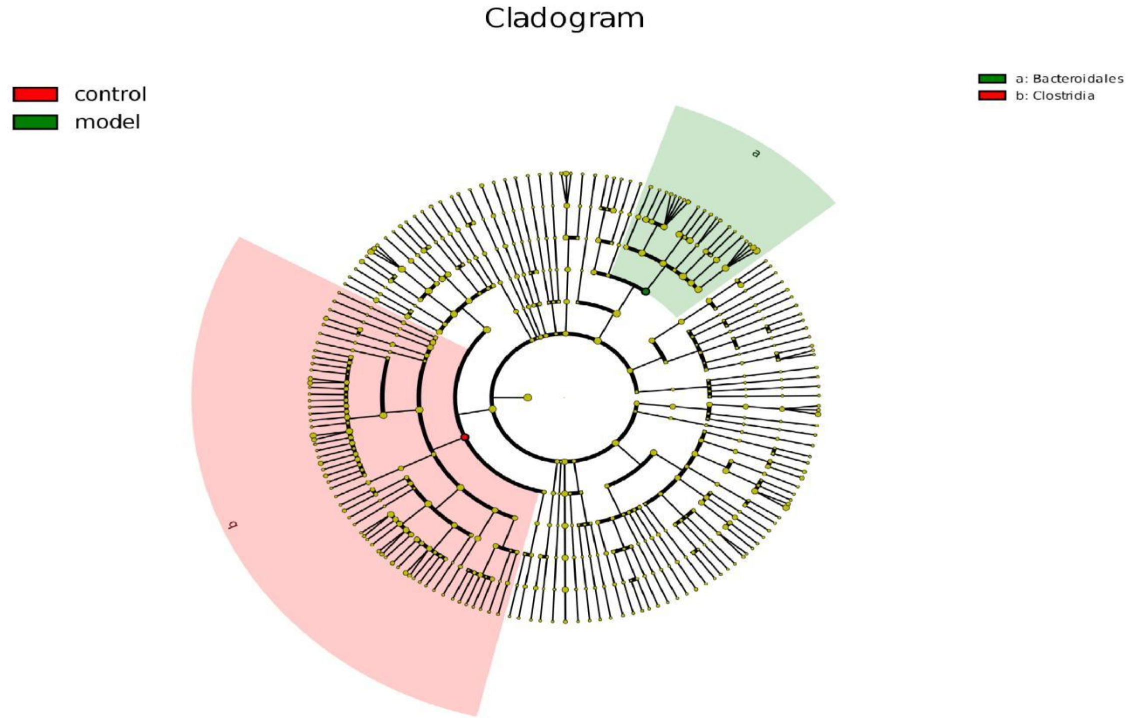

B

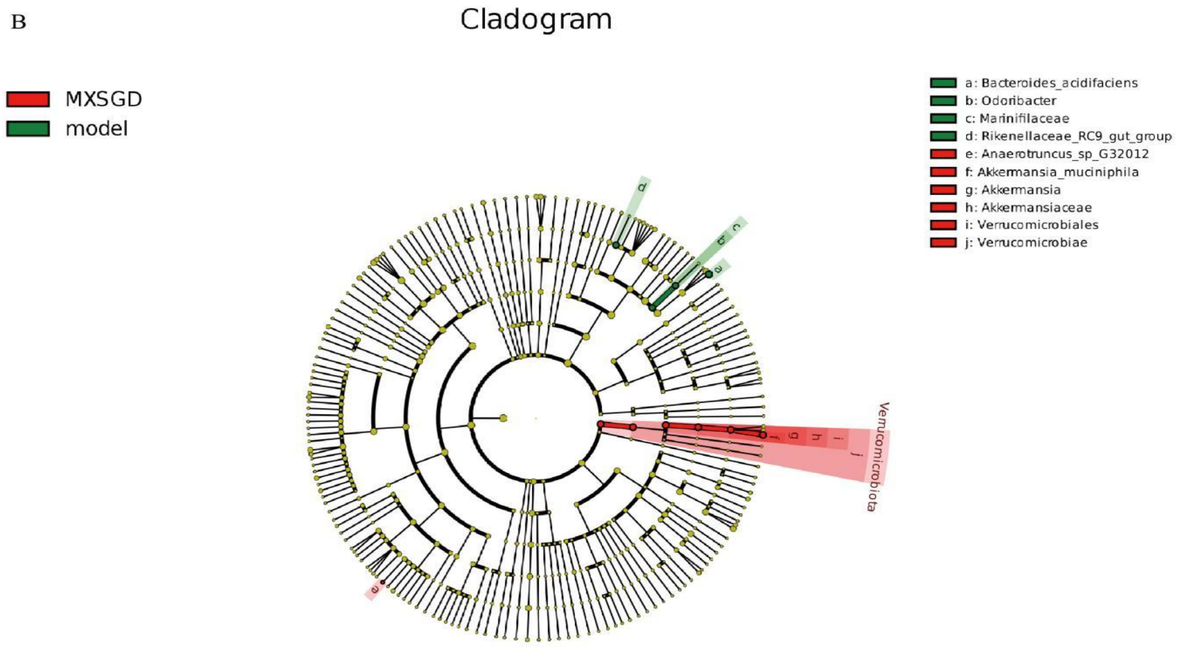

Fig.3 MXSGD Regulates the Diversity and Composition of the Gut Microbiota

(**A**): LEfSe analysis, control VS model ; (**B**): LEfSe analysis, MXSGD VS model

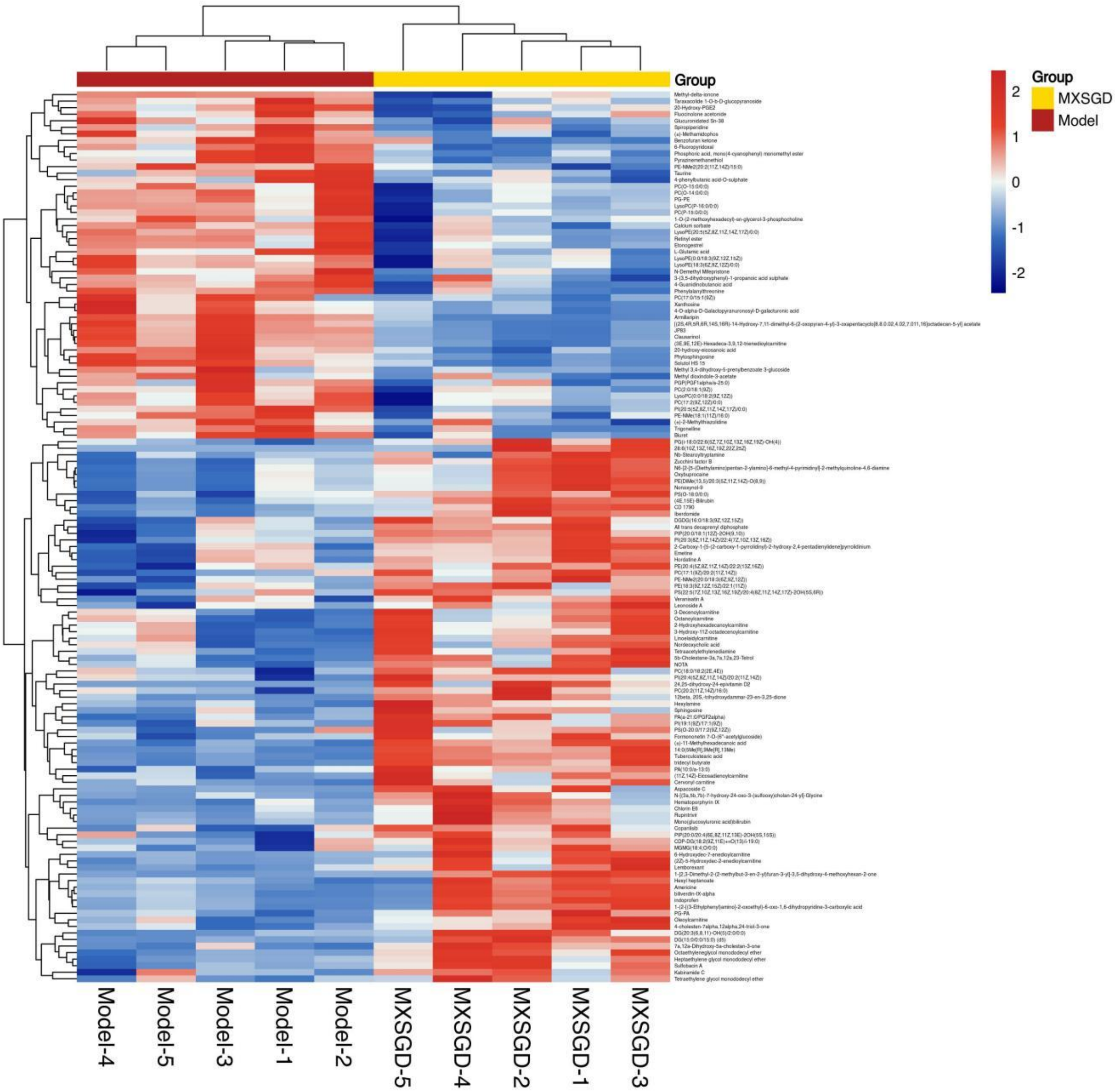

A

Fig. 4   Differential metabolites

(**A**):MXSGD VS model; Differential metabolite heat map, the color from blue to red indicates that the expression abundance of metabolites is from low to high; (n=5).

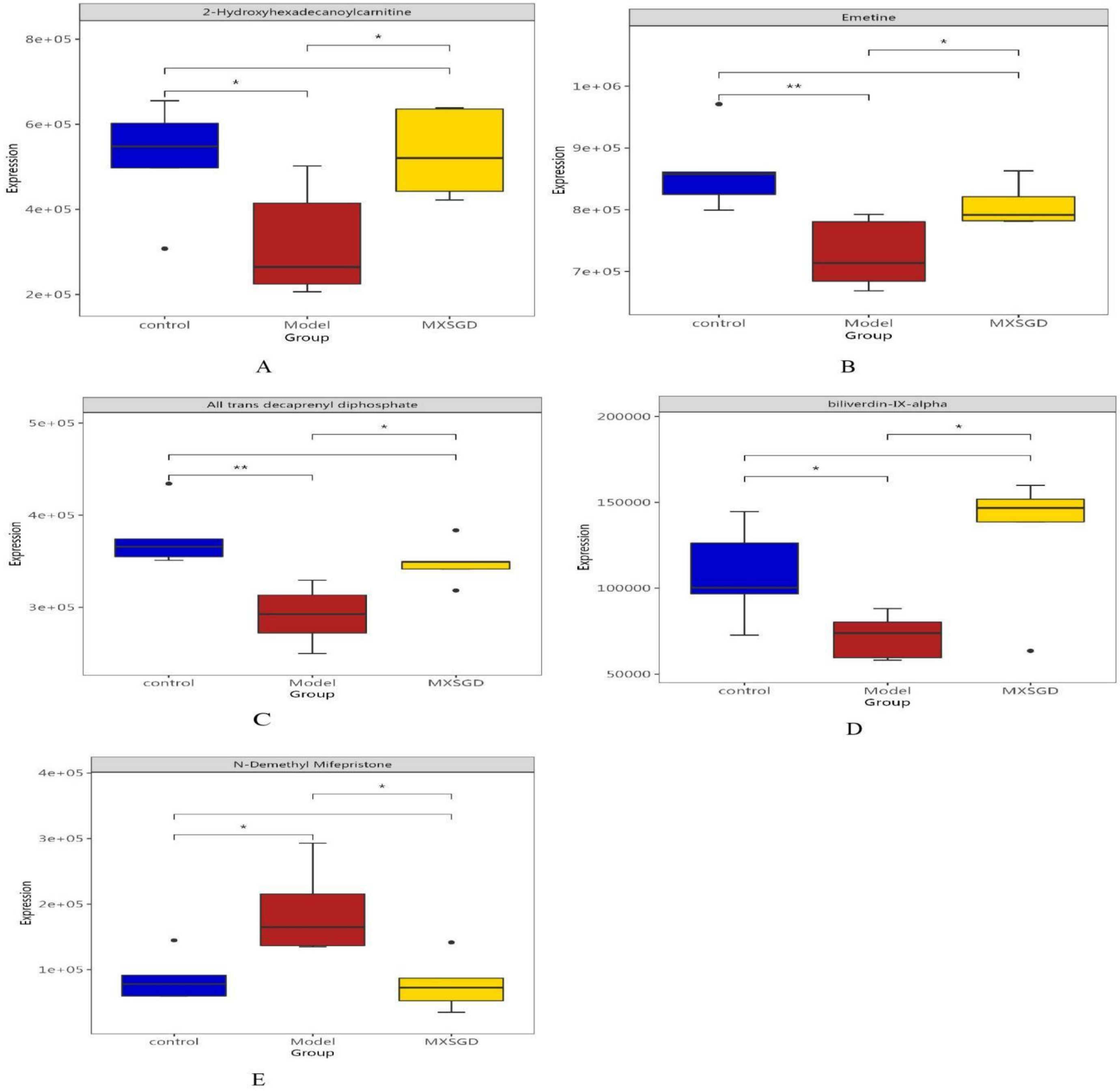

Fig. 5 Significant difference metabolites

(**A**): 2-Hydroxyhexadecanoy lcarnitine; (**B**)Emetine; (**C**):All trans decaprenyl diphosphate; (**D**):Biliverdin-IX-alpha; (**E**):N-demethyl Mifepristone metabolite;(n=5).

A

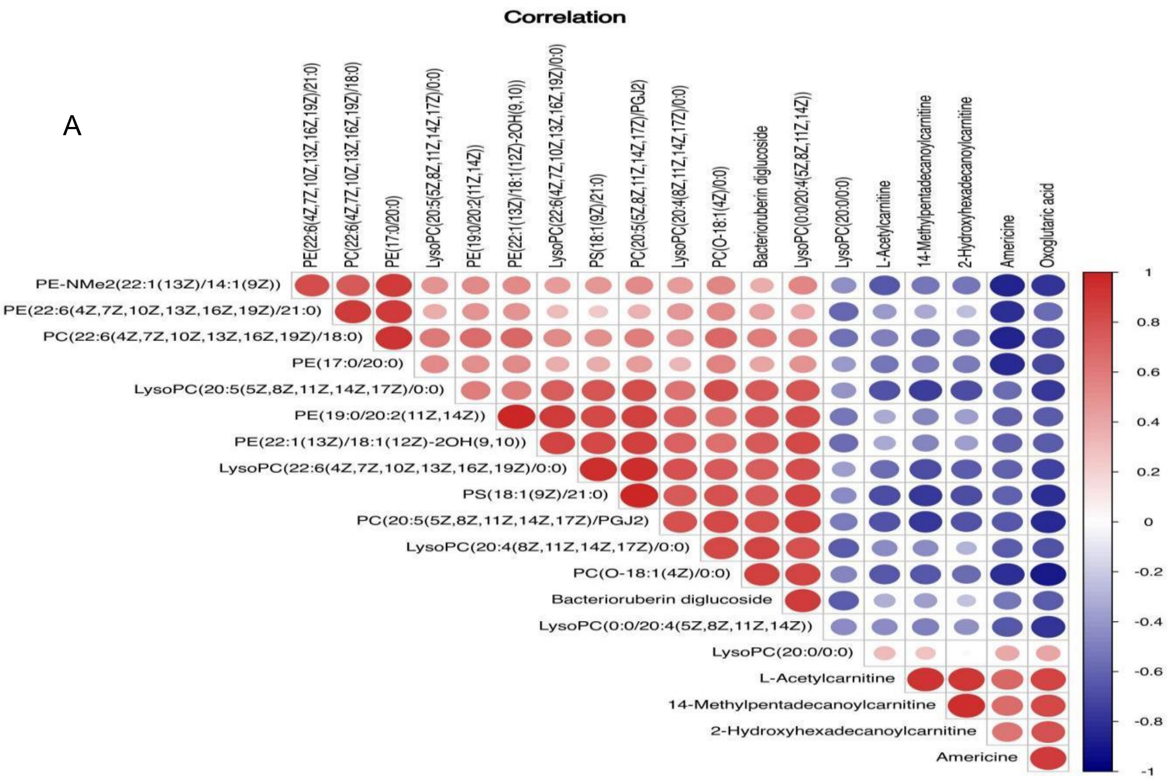

Correlation
PE(22:6(4Z,7Z,10Z,13Z,16Z,19Z)/21:0)
PC(22:6(4Z,7Z,10Z,13Z,16Z,19Z)/18:0)
PE(17:0/20:0)
LysoPC(20:5(5Z,8Z,11Z,14Z,17Z)/0:0)
PE(19:0/20:2(11Z,14Z))
PE(22:1(13Z)/18:1(12Z)-2OH(9,10))
LysoPC(22:6(4Z,7Z,10Z,13Z,16Z,19Z)/0:0)
PS(18:1(9Z)/21:0)
PC(20:5(5Z,8Z,11Z,14Z,17Z)/PGJ2)
LysoPC(20:4(8Z,11Z,14Z,17Z)/0:0)
PC(O-18:1(4Z)/0:0)
Bacterioruberin diglucoside
LysoPC(0:0/20:4(5Z,8Z,11Z,14Z))
LysoPC(20:0/0:0)
L-Acetylcarnitine
14-Methylpentadecanoylcarnitine
2-Hydroxyhexadecanoylcarnitine
Americine
Oxoglutaric acid
PE-NMe2(22:1(13Z)/14:1(9Z))
PE(22:6(4Z,7Z,10Z,13Z,16Z,19Z)/21:0)
PC(22:6(4Z,7Z,10Z,13Z,16Z,19Z)/18:0)
PE(17:0/20:0)
LysoPC(20:5(5Z,8Z,11Z,14Z,17Z)/0:0)
PE(19:0/20:2(11Z,14Z))
PE(22:1(13Z)/18:1(12Z)-2OH(9,10))
LysoPC(22:6(4Z,7Z,10Z,13Z,16Z,19Z)/0:0)
PS(18:1(9Z)/21:0)
PC(20:5(5Z,8Z,11Z,14Z,17Z)/PGJ2)
LysoPC(20:4(8Z,11Z,14Z,17Z)/0:0)
PC(O-18:1(4Z)/0:0)
Bacterioruberin diglucoside
LysoPC(0:0/20:4(5Z,8Z,11Z,14Z))
LysoPC(20:0/0:0)
L-Acetylcarnitine
14-Methylpentadecanoylcarnitine
2-Hydroxyhexadecanoylcarnitine
Americine
1
0.8
0.6
0.4
0.2
0
-0.2
-0.4
-0.6
-0.8
-1

B

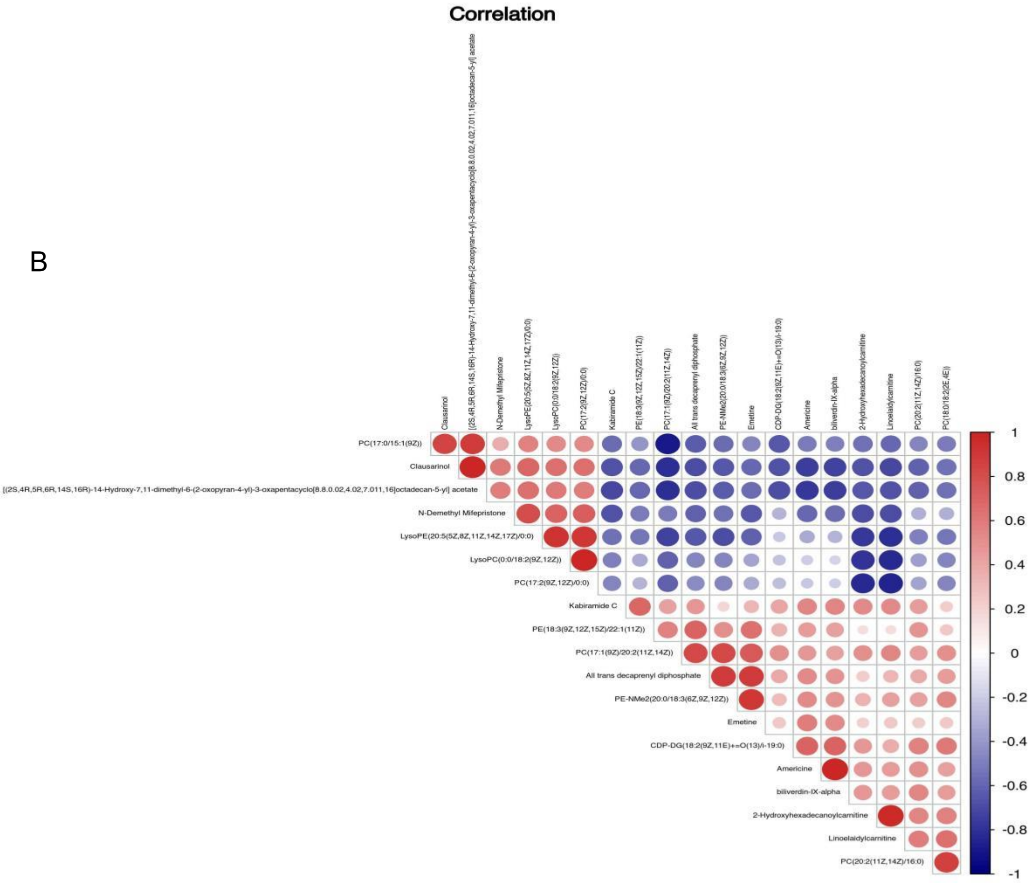

Correlation
Clausarinol
N-Demethyl Mifepristone
LysoPE(20:5(5Z,8Z,11Z,14Z,17Z)/0:0)
LysoPC(0:0/18:2(9Z,12Z))
PC(17:2(9Z,12Z)/0:0)
Kabiramide C
PE(18:3(9Z,12Z,15Z)/22:1(11Z))
PC(17:1(9Z)/20:2(11Z,14Z))
All trans decaprenyl diphosphate
PE-NMe2(20:0/18:3(6Z,9Z,12Z))
Emetine
CDP-DG(18:2(9Z,11E)+=O(13)/i-19:0)
Americine
biliverdin-IX-alpha
2-Hydroxyhexadecanoylcarnitine
Linoelaidylcarnitine
PC(20:2(11Z,14Z)/16:0)
PC(18:0/18:2(2E,4E))
PC(17:0/15:1(9Z))
Clausarinol
[(2S,4R,5R,6R,14S,16R)-14-Hydroxy-7,11-dimethyl-6-(2-oxopyran-4-yl)-3-oxapentacyclo[8.8.0.02,4.02,7.011,16]octadecan-5-yl] acetate
N-Demethyl Mifepristone
LysoPE(20:5(5Z,8Z,11Z,14Z,17Z)/0:0)
LysoPC(0:0/18:2(9Z,12Z))
PC(17:2(9Z,12Z)/0:0)
Kabiramide C
PE(18:3(9Z,12Z,15Z)/22:1(11Z))
PC(17:1(9Z)/20:2(11Z,14Z))
All trans decaprenyl diphosphate
PE-NMe2(20:0/18:3(6Z,9Z,12Z))
Emetine
CDP-DG(18:2(9Z,11E)+=O(13)/i-19:0)
Americine
biliverdin-IX-alpha
2-Hydroxyhexadecanoylcarnitine
Linoelaidylcarnitine
PC(20:2(11Z,14Z)/16:0)
1
0.8
0.6
0.4
0.2
0
-0.2
-0.4
-0.6
-0.8
-1

Fig.6 Multivariate statistical analysis of serum metabolite distribution

(**A**):Correlation analysis of TOP-20 differential metabolites between model and control; (**B**):Correlation analysis of TOP-20 differential metabolites between MXSGD and model.(n=5)
